# From Theory to Clinical Practice: Assessing the Knowledge of Medical Staff Working in Gynecology and Obstetrics Departments and Medical Students Regarding the Diagnosis and Treatment of Postpartum Depression

**DOI:** 10.7759/cureus.91881

**Published:** 2025-09-09

**Authors:** Urszula Michalik-Marcinkowska, Magdalena Rduch, Monika Zaborska, Zuzanna Niedbal, Klaudia Bogdan, Urszula Janicka, Weronika Ogonowska

**Affiliations:** 1 Department of Family Medicine and Public Health, University of Opole, Opole, POL; 2 Faculty of Nursing, College of Applied Sciences, Ruda Śląska, POL

**Keywords:** doctors, medical knowledge, medical staff education, medicine students, midwifes, nurses, postnatal care, postpartum depression

## Abstract

Introduction

The aim of this study was to evaluate the knowledge of medical staff and final-year medical students regarding current guidelines for the diagnosis and treatment of postpartum depression.

Methods

The study involved two groups of participants, totaling 460 individuals: active healthcare professionals working with pregnant and postpartum women and their families, and 5th- and 6th-year medical students. Participants were selected using a snowball sampling method. An author-designed questionnaire, based on the Edinburgh Postnatal Depression Scale (EPDS), consisting of 17 items, was used to assess participants' knowledge. One point was awarded for each correct response, and no points were given for incorrect responses. The maximum possible score was 17.

Results

The mean score on the knowledge test was 11 out of 17 points. Among all respondents, 165 (36%) demonstrated an average level of knowledge about postpartum depression, while 294 (63.7%) exhibited a high level of knowledge. Medical students who had completed courses in gynecology, obstetrics, and psychiatry achieved the highest scores (p < 0.01). Medical professionals with experience in managing postpartum depression rated the support they provided during medical care as 4.8 on a 1-10 scale.

Conclusion

Approximately half of the respondents were familiar with the definition of postpartum depression and demonstrated a competent understanding of its diagnostic criteria. Professional factors such as work experience and formal education did not appear to significantly differentiate the level of knowledge about postpartum depression. The highest knowledge scores were observed among medical students who had completed coursework in gynecology, obstetrics, and psychiatry, rather than among practicing medical professionals.

## Introduction

Postpartum mental disorders include maternity blues (postpartum blues, PB), postpartum depression (PPD), puerperal psychosis (PP), and postpartum hypomania (PH) [1].

In the International Classification of Diseases, Eleventh Revision (ICD-11) classification [2], PPD is included in the category of mental and behavioral disorders associated with pregnancy, childbirth, and the puerperium, not elsewhere classified [2]. This category is defined as "a syndrome associated with pregnancy or the puerperium (beginning within about six weeks of delivery) that includes significant mental and behavioral features but does not meet the diagnostic requirements of any specific mental and behavioral disorder” and can be distinguished as clinical symptoms with or without psychotic features [2].

The main criteria for diagnosing a major depressive episode are depressed mood and anhedonia, i.e., loss of pleasure in previously performed activities, lasting at least two weeks [3]. PPD is characterized by a clinical triad of symptoms, such as low self-esteem, hypochondria, and tension, which are not typical features of a severe or moderate depressive episode [4].

A meta-analysis of 565 studies from 80 countries and regions by Wang et al. indicated a prevalence of PPD among 17.22% of women worldwide [5]. However, these data likely underestimate the true prevalence due to underdiagnosis [6]. Young mothers often seek help late or not at all, primarily because of fear of negative evaluation by loved ones and stereotypical perceptions of women [7]​ in the early postpartum period. Women's behaviors during this time are sometimes described as "unstable" or even "hysterical," leading to suppression of feelings and symptoms that could indicate the developing depression [8].

Disturbances in sleep, eating, concentration, and attention are common during disorders, and adaptation to motherhood can further hinder diagnosis. The challenge is compounded by multiple complex risk factors, including a wide range of socioeconomic and health conditions [9], as well as pregnancy and delivery-related factors [10], with particular emphasis on cesarean section [11]. Additional important predictors are included in the Postpartum Depression Predictors Inventory [12]. Awareness of PPD risk and knowledge of diagnostic and treatment procedures among medical staff also play a crucial role [13]. Currently, 12 instruments combined with prenatal, perinatal, and postnatal risk factors are used to assess the occurrence of PPD [14]. The most widely used and validated tool is the Edinburgh Postnatal Depression Scale (EPDS) [15]. Although not a perfect diagnostic instrument [16], it is considered the gold standard for PPD screening [14]. The EPDS consists of 10 questions, each scored from 0 to 3. There is no consensus among clinicians regarding the cut-off score for diagnosing PPD; both 10 and 13 points are commonly used [17].

Risk assessment is typically conducted by midwives, nurses, and gynecologists, although psychiatrists and psychologists should also be included in diagnostic and educational teams. According to Polish standards of perinatal care, a midwife or doctor providing care to a pregnant woman should assess the risk and severity of depression symptoms twice during pregnancy, between the 11th-14th and 33rd-37th weeks, and once within a month postpartum during a community midwife visit [18]. These assessments require up-to-date scientific knowledge among medical staff. To date, no national assessment of such knowledge has been conducted in Poland. Existing literature focuses mainly on midwives and nurses, with limited attention to doctors or medical students. However, physicians working with pregnant women and in the preconception period are in a key position to educate women and their families about preventing PPD, potentially yielding the most positive outcomes [19].

Objectives

To assess the knowledge of medical staff and medical students in their final two years regarding current guidelines for diagnosing and treating PPD. To identify personal and professional factors influencing the level of awareness among doctors, midwives, and nurses.

## Materials and methods

Participants

Two groups were included in the study: active medical professionals (doctors, midwives, and nurses) working with pregnant and postpartum women and their families, and 5th- and 6th-year medical students who had completed coursework in gynecology, obstetrics, and psychiatry, disciplines in which the curricula are expected to include content on postpartum depression. In Poland, undergraduate medical education lasts six years.

The groups consisting of 460 people were studied. Table 1 presents the group characteristics.

**Table 1:** Participants' characteristics.

Personal and professional factors	N (%)
Gender	Women: 385 (84)
	Men: 75 (16)
Place of living	City: 354 (77)
	Village:106 (23)
Level of education	Medium: 203 (44)
	High: 257 (56)
Profession (medical staff)	Midwives: 64 (14)
	Nurses: 124 (27)
	Doctors: 72 (16)
Profession (students)	5^th^ year of medicine: 134 (29)
	6^th^ year of medicine: 66 (14)
Specialization (gynecology, psychiatry, or obstetrics)	Yes: 129 (28)
	No: 331 (72)
Having children	Yes: 187 (41)
	No: 273 (59)
Average age of respondents	31.48 years (SD = 10.43); min = 24, max = 62
Average length of service	7.26 years (SD =10.22); min = 1 year, max = 38 years

The following inclusion criteria were used: being an active health professional, i.e., a doctor, midwife, or nurse working in gynecology and obstetrics departments or in the community with pregnant and postpartum women; medical students after at least the 5th year, having completed courses in gynecology, obstetrics, and psychiatry; completion of coursework covering pre- and postnatal care principles; and participants providing informed consent to participate.

The following criteria were applied to exclude participants from the study: absence of informed consent to participate in the study; physicians who are not currently engaged in active professional practice; healthcare professionals who do not provide direct clinical care or caregiving services to pregnant or postpartum women; medical students who have not completed at least the fifth year of their studies; students who have not completed the mandatory courses in gynecology, obstetrics, and psychiatry; questionnaires that were incomplete or incorrectly completed; data obtained from questionnaires or surveys of insufficient completeness or inadequate quality, precluding reliable statistical analysis.

Data sources/measurement

A proprietary 20-question survey (17 on knowledge, three on perinatal care standards) and demographic/professional metrics were used. The EPDS and the latest perinatal care recommendations were analyzed to develop questions on the definition, risk factors, prevalence, distinguishing features, and therapeutic methods for postpartum depression. All questions were closed-ended. One point was awarded for each correct answer (max = 17 points). Knowledge levels were assessed as follows: low (0-6 points), medium (7-12 points), and high (13-17 points).

The questionnaire underwent a pilot test with 20 medical professionals (14 midwives, six doctors). No content-related comments were received. A repeated survey yielded Cronbach's alpha of 0.87.

Setting

The survey was conducted electronically. A link containing the survey was created and shared on social networks for medical students from all over Poland, as well as on nationwide groups for medical personnel. A snowball method was used in the selection of respondents. The selection was therefore purposive and random. The survey was conducted in 2023, covering 10 voivodeships.

In the instructions for completing the questionnaire, the respondents were informed about the time to complete the survey. It was estimated at 15 minutes. After this time, the survey was closed and ended with the question that the person answered at the time of closing. The time limitation was intended to serve the purpose of providing answers that were derived from the respondents' own knowledge and experience, and to eliminate the potential use of internet sources when answering.

Statistics

For statistical analysis, Statistica version 13.3 (TIBCO Software Inc., Palo Alto, CA) was used. Results were accumulated in Microsoft Excel (Microsoft Corporation, Redmond, WA). Firstly, descriptive statistics were calculated: counts, arithmetic mean, standard deviation, and median. After checking normality by the Shapiro-Wilk test, nonparametric Mann-Whitney U (for two variables) or Kruskal-Wallis (for three variables) tests were used to identify the significance of intergroup differences. Spearman's rho test was used for measuring the strength of association between two variables. To assess the conformity of the distribution of random variables with the normal distribution, the Pearson chi-squared test of independence was applied, and the strength of association between variables was evaluated using the phi (φ) coefficient. The statistical significance at p ≤ 0.05 was adopted.

Ethics

The Bioethics Committee of the University of Opole officially issued permission to conduct the research (L.dz. EZD/213292/2023).

## Results

The average knowledge score among all respondents was 11 points (SD = 1.72; min = 4; max = 6; median = 11). One participant scored the lowest score (4 points), and five people obtained the highest score (16 points). The most frequent score was 13 points, obtained by 122 respondents, while only one respondent scored 4 points. Table 2 presents results from the PPD knowledge test. Taking into account that the only statistically significant factor was gender, among the study participants, women scored higher in the test on the diagnosis and treatment of PPD.

**Table 2:** Results of the postpartum depression knowledge test regarding personal and professional factors.

Personal factor	N (%)	Mean score (SD); min-max	Mann-Whitney U-test (U; Z; p)
Gender	Women: 385 (84)	12.20 (1.88); 4-16	11540.50; 2,75; p<0.01
	Men: 75 (16)	11.48 (2.13); 7-15	11540.50; 2,75; p<0.01
Place of living	City: 354 (77)	12.09 (1.92): 4-16	23606.50; -1.75; p>0.05
	Village: 106 (23)	12.10 (1.90); 8-15	23606.50; -1.75; p>0.05
Level of education	Medium: 203 (44)	12.12 (2.01); 4-16	17377.00; 0.72; p>0.05
	High: 257 (56)	12.05 (2.01); 7-16	17377.00; 0.72; p>0.05
Profession	Medical staff: 260 (57)	12.16 (1.84); 7-16	58942.50; 0.69; p>0.05
	Students of medicine: 200 (43)	11.97 (2-05); 4-16	58942.50; 0.69; p>0.05
Specialization (gynecology, psychiatry, or obstetrics)	Yes: 129 (28)	12.21 (1.83); 7-16	28848.00; 0.69; p>0.05
	No: 331 (72)	12.03 (1.98); 4-16	28848.00; 0.69; p>0.05
Having children	Yes: 187 (41)	12.20 (1.94); 4-16	24955.50; -0.40; p>0.05
	No: 273 (59)	12.00 (1.93); 6-16	24955.50; -0.40; p>0.05

Table 3 divides the results into two levels of knowledge: medium (6-11 points) and high (12-16 points). Among all respondents, 166 (36%) people had a medium level of knowledge about PPD, while 294 (64%) people had a high level of knowledge.

**Table 3:** The level of knowledge of respondents regarding personal and professional factors.

Personal factor	N (%)	Level of knowledge		Statistics chi^2^ Pearson-test (chi^2^; df; p; phi)
		Medium (6-11 points)	High (12-16 points)	
Gender	Women: 385 (84)	128 (33.25)	257 (66.75)	8.43; 1; p<0.01; 0.135
	Men: 75 (16)	38 (50.67)	37 (49.33)	8.43; 1; p<0.01; 0.135
Place of living	City: 354 (77)	129 (36.44)	225 (63.56)	0.18; 1; p>0.05
	Village: 106 (23)	37 (34.91)	69 (65.09)	0.18; 1; p>0.05
Level of education	Medium: 203 (44)	69 (33.99)	134 (66.01)	0.81; 1; p>0.05
	High: 257 (56)	97 (37.74)	160 (62.26)	0.81; 1; p>0.05
Profession	Medical staff: 260 (57)	93 (35.77)	167 (64.23)	0.00; 1; p>0.05
	Student: 200 (43)	73 (36.50)	127 (63,50)	0.00; 1; p>0.05
Specialization (gynecology, psychiatry, or obstetrics)	Yes: 129 (28)	42 (32.56)	87 (67.44)	0.89; 1; p>0.05
	No: 331 (72)	124 (37.46)	207 (62.54)	0.89; 1; p>0.05
Having children	Yes: 187 (41)	61 (32.62)	126 (67.38)	1.84; 1; p>0.05
	No: 273 (59)	105 (38.46)	168 (61.54)	1.84; 1; p>0.05

Additionally, the Kruskal-Wallis test was used to analyze the statistical relationship between knowledge and respondents' professions. A statistically significant difference was observed: H(4, N = 460) = 15.82; p < 0.01. The mean number of correct answers was 11.08 (SD = 1.98) among doctors, 11.02 (SD = 1.31) among midwives, 10.86 (SD = 1.81) among nurses, 10.41 (SD = 1.79) among students who had completed a course in gynecology and obstetrics, and 11.59 (SD = 1.50) among students who had completed classes in gynecology, obstetrics, and psychiatry. In the case of knowledge levels (medium-high), no statistically significant differences were observed.

The Spearman rank test indicated no statistically significant correlation between the age or work experience of the respondents and the overall score of correct answers.

Table 4 shows the percentage of correct and incorrect answers given by the respondents. The most incorrect answers concerned knowledge of the definition of PPD, first-line treatment, and answers to the questions whether PPD goes away on its own.

**Table 4:** Correct and incorrect answers from the knowledge test (N = 460). ICD-11: International Classification of Diseases, Eleventh Revision.

Test question	Correct answer	Number of correct answers, N (%)	Number of incorrect answers, N (%)
Please provide the correct definition of postpartum depression according to the latest ICD-11 classification.	A syndrome associated with pregnancy or the postpartum period (beginning within approximately 6 weeks after delivery) that includes significant mental and behavioral characteristics but does not meet the diagnostic requirements for any specific mental or behavioral disorders.	216 (47)	244 (53)
What time criterion determines postpartum depression?	Persistence of depression symptoms for more than 2 weeks	322 (70)	138 (30)
What percentage of women suffer from postpartum depression?	10-22%	288 (62)	172 (38)
Do pregnancy complications, including the occurrence of premature contractions, bleeding, and urinary tract and vaginal infection, increase the risk of emotional disorders during the postpartum period?	Yes	433 (94)	27 (6)
Does postpartum sadness, known as "baby blues," always lead to postpartum depression?	No	417 (90)	43 (10)
Do personality traits influence the development of postpartum depression?	Yes	433 (94)	27 (6)
Can excessive concern for the child's life and health be a symptom of postpartum depression?	Yes	356 (77)	104 (23)
What is the name of the scale that is the most common tool for early detection of postpartum mood disorders?	Edinburgh Postnatal Depression Scale	389 (85)	71 (15)
What is the maximum number of points that can be scored on the Edinburgh Postnatal Depression Scale?	30 points	346 (75)	114 (25)
What score on the Edinburgh Postnatal Depression Scale, according to the Polish Association of Midwives, is the specialist's recommendation?	10 points and more	336 (73)	124 (27)
Does a positive result (1, 2, or 3 points) in response to question 10 of the Edinburgh Postnatal Depression Scale regarding the desire to harm oneself require immediate medical consultation?	Yes	420 (91)	40 (9)
Is pharmacotherapy used to treat postpartum depression?	Yes	441 (96)	19 (4)
What is the first treatment for postpartum depression?	Psychotherapy	209 (45)	251 (55)
Can postpartum depression turn into depression?	Yes	453 (98)	7 (2)
The most common symptoms of postpartum depression include anxiety, irritability, difficulty thinking and memory, fatigue, insomnia, anxiety, guilt, suicidal thoughts, and anhedonia. What do you think this term means?	Anhedonia - not feeling pleasure in situations that are pleasant; characteristic of depression and other mental disorders	353 (77)	107 (23)
Can postpartum depression go away on its own?	No	144 (31)	316 (69)

Moreover, in the group of respondents, 108 people declared that during their work, they had dealt with a woman diagnosed with PPD. Medical staff who dealt with a woman suffering from PPD rated the level of support they provided while providing medical services as 4.8 on a 1-10 points scale (where 1 means the support was insufficient, and 10 means totally sufficient; SD = 7.15; median = 5; min = 1; max = 10). The respondents rated their self-evaluated knowledge about PPD at 4.64 (SD = 1.96; median = 5) on a 1-10 points scale (where 1 means the knowledge was insufficient, and 10 means totally sufficient). Unfortunately, only 42 (9.1%) medical staff and medicine students stated that Polish standards of perinatal care are sufficient to provide women with immediate psychological care.

We also asked respondents who should primarily care for a woman suffering from PPD. Surprisingly, the answers were not obvious. Among the studied people, 171 (37%) stated that care should be provided by a psychologist, 154 (33%) stated by a psychiatrist, and 115 (25%) stated by husband/partner/member of the closest family. Only 10 (2%) respondents indicated doctors, and nine (2%) mentioned midwives as frontline assistance.

## Discussion

The study revealed that while 64% of respondents demonstrated high knowledge of PPD, 36% scored at a medium level. Over half could not correctly define the disorder, and 15% did not know the primary diagnostic tool. Similar deficits were noted by Fiavor et al. [20], where most nurses and midwives did not educate mothers about PPD symptoms. Some respondents in our study admitted they did not educate women because it was not formally part of their duties [13].

Qualitative research from Iran confirms that medical staff face difficulties diagnosing PPD due to insufficient knowledge and the subtle nature of symptoms [13]. Link et al. [21] also reported low self-efficacy in PPD assessment among students and nurses.

In contrast, general practitioners in countries like Ireland and England expressed confidence in diagnosing perinatal depression but reported stigma concerns, time constraints, and reliance on personal experience rather than current literature [22]. They also showed willingness to participate in further training [23].

Although nursing, midwifery, and medical students in Poland receive health education courses, these often focus on theory rather than practical skills. Introducing PPD into medical curricula is essential for improving professional competence [24]. However, education is not the only barrier. Polish mental health services face resource limitations [24], and fewer than 10% of our respondents considered the current perinatal care standards as sufficient.

The absence of national guidelines for prevention, treatment, and follow-up for parents with PPD, combined with the lack of integration with mental health services [25], contributes to underdiagnosis. While integrated intervention models exist elsewhere [23], they are not implemented in Poland. This results in discrepancies between screening guidelines and practice, particularly regarding tools, staff qualifications, and referral pathways [26].

Another challenge is women’s reluctance to seek help, often due to isolation, guilt, and societal expectations of motherhood [27-29]. These factors can impede both perinatal [28] and postnatal [29] depression care.

Our study did not include psychologists, despite their central role in therapy. Cognitive-behavioral therapy has shown greater efficacy than pharmacotherapy in PPD treatment, especially when initiated during pregnancy and led by trained specialists. Yet, over half of our participants were unaware that psychotherapy, not pharmacotherapy, is the first-line treatment.

## Conclusions

Although only half of the respondents could define PPD, most were familiar with its diagnostic criteria. Professional factors, such as work experience and education, did not differentiate the level of awareness about PPD. The highest knowledge scores were observed among medical students who had already received training in gynecology, obstetrics, and psychiatry during their studies, rather than among medical staff.

Interestingly, the highest scores were achieved not by practicing staff, but by medical students who had completed both gynecology/obstetrics and psychiatry courses. Only 10% of professionals working in perinatal care considered the current Polish standards sufficient for providing adequate psychological support. At the same time, psychological and psychiatric support is indicated as the most preferred. As a result, the situation is complicated: access to psychological support in maternity wards is very limited, and the diagnosis of PPD is dependent on the awareness, sensitivity, and knowledge of doctors and midwives, which determines whether a woman and her family will receive adequate help.
